# *Nr2e3* is a genetic modifier that rescues retinal degeneration and promotes homeostasis in multiple models of retinitis pigmentosa

**DOI:** 10.1038/s41434-020-0134-z

**Published:** 2020-03-02

**Authors:** Sujun Li, Shyamtanu Datta, Emily Brabbit, Zoe Love, Victoria Woytowicz, Kyle Flattery, Jessica Capri, Katie Yao, Siqi Wu, Michael Imboden, Arun Upadhyay, Rasappa Arumugham, Wallace B. Thoreson, Margaret M. DeAngelis, Neena B. Haider

**Affiliations:** 1 Department of Ophthalmology, Schepens Eye Research Institute, Massachusetts Eye and Ear, Harvard Medical School, Boston, MA USA; 2 Ocugen Inc., Malvern, PA USA; 3 Department of Ophthalmology and Visual Sciences, Truhlsen Eye Institute, University of Nebraska Medical Center, Omaha, NE USA; 4 Moran Eye Center for Translational Medicine, Salt Lake, UT USA

**Keywords:** Gene therapy, Transcription, Gene regulation

## Abstract

Recent advances in viral vector engineering, as well as an increased understanding of the cellular and molecular mechanism of retinal diseases, have led to the development of novel gene therapy approaches. Furthermore, ease of accessibility and ocular immune privilege makes the retina an ideal target for gene therapies. In this study, the nuclear hormone receptor gene *Nr2e3* was evaluated for efficacy as broad-spectrum therapy to attenuate early to intermediate stages of retinal degeneration in five unique mouse models of retinitis pigmentosa (RP). RP is a group of heterogenic inherited retinal diseases associated with over 150 gene mutations, affecting over 1.5 million individuals worldwide. RP varies in age of onset, severity, and rate of progression. In addition, ~40% of RP patients cannot be genetically diagnosed, confounding the ability to develop personalized RP therapies. Remarkably, *Nr2e3* administered therapy resulted in reduced retinal degeneration as observed by increase in photoreceptor cells, improved electroretinogram, and a dramatic molecular reset of key transcription factors and associated gene networks. These therapeutic effects improved retinal homeostasis in diseased tissue. Results of this study provide evidence that *Nr2e3* can serve as a broad-spectrum therapy to treat multiple forms of RP.

## Introduction

Recent studies have demonstrated the potential of gene therapy to attenuate or slow the progression of previously untreatable inherited diseases [1]. Gene therapy has evolved from concept to clinic for various genetic disorders including hematological [2], immunological [3], ocular [4, 5], neurodegenerative [6], and metabolic disorders [7]. Due to its ease of accessibility and immune privilege, the eye holds potential as an ideal organ for gene therapy. The most widely accepted success of adeno-associated virus (AAV) vector-mediated ocular gene replacement is for treatment of Leber’s congenital amaurosis 2 (LCA2), a rare retinal disease due to mutations in the *RPE65* gene [8, 9]. Currently, there are several ongoing clinical gene augmentation trials for other rare inherited retinal diseases [10–13]. However, several factors such as gene size, gene function, and the large number (~40%) of retinitis pigmentosa (RP) patients that cannot be genetically diagnosed present challenges for developing individual gene replacement/augmentation-based therapies. Thus, new therapeutic approaches are needed to circumvent these limitations. This study evaluates a unique approach using the nuclear hormone receptor (NHR) gene *Nr2e3* as a genetic modifier and therapeutic agent to treat multiple retinal degenerative diseases. Results of this study demonstrate the power of a single genetic modifier in treating retinal diseases.

RP represents a group of inherited diseases, affecting an estimated 1 in 4000 individuals, that cause degeneration of rod and cone photoreceptor cells, leading to the severe vision loss [14, 15]. RP can be inherited through multiple modes of inheritance such as autosomal dominant (30–40% of cases), autosomal recessive (50–60% of cases), or X-linked (5–15% of cases) manner in syndromic or nonsyndromic forms [16–18]. Over 150 unique gene mutations have been associated with RP, making it highly heterogenic, with high variability in disease onset, severity, and progression [19–23].

Genetic modifiers are defined as allelic variants found within the normal population [24, 25]. Modifier genes can significantly affect disease outcomes, impacting onset, rate of progression, and severity [24, 26, 27]. Genetic modifier genes are powerful modulators that can enhance or suppress disease phenotypes [24, 27–29]. The direct impact of genetic modifiers has been studied extensively in several diseases including cystic fibrosis, epileptic encephalopathy, spinocerebellar ataxia type 1, spinal muscular atrophy, dystonia, and retinal degeneration where drastically altered phenotypes occur when genetic background is shifted [30–38]. Haider et al. discovered that shifting the *rd7* mutation, a recessive mutation in NHR 2 family e, member 3, *Nr2e3* that results in slow progressive retinal degeneration, onto three different genetic backgrounds resulted in complete suppression of the *rd7* phenotype in all strains evaluated, and genetic mapping revealed that several modifier genes could independently account for this suppression [39]. The NHR 1 family d, member 1 (*Nr1d1)*, a NHR gene, and cofactor of *Nr2e3*, was identified as one of the genetic modifiers that can ameliorate *Nr2e3* associated retinal degeneration [40].

Mutations in human *NR2E3* are associated with several forms of retinal degeneration that vary in phenotype and were categorized by their clinical diagnosis as they were discovered. These clinical categories include the recessive diseases enhanced S-cone syndrome (ESCS), Goldmann-Favre syndrome (GFS), and clumped pigmentary retinal degeneration (CPRD) [41–43]. *NR2E3* mutations are also associated with up to 1% of all autosomal dominant retinitis pigmentosa (adRP) [44, 45]. The association of *NR2E3* with several clinical phenotypes and varying modes of inheritance strongly indicates that these retinal diseases manifest on a permissive or selective genetic background and are influenced, at least in part, by genetic modifier genes [46–49]. Given the role of NHRs such as *NR2E3*, to modulate numerous key biological networks essential for maintaining retinal homeostasis, this study evaluated *Nr2e3* as a broad-spectrum genetic modifier with the potential to attenuate retinal degeneration in several different mouse models.

In this study, the efficacy of subretinal delivery of AAV8-*Nr2e3* to attenuate and ameliorate retinal degeneration was assessed in five independent RP models that represent the heterogeneity observed in human RP disease. The five RP models tested were FVB-*Pde6ß* ^*rd1*^/NJ (*rd1*), Rhodopsin null allele (*Rho*^−/−^), B6.129S6(Cg)-*Rho*^*tm1.1Kpal*^/J (*Rho*^*P23H*^), BXD24/TyJ-*Cep290*^*rd16*^/J (*rd16*) and *Nr2e3*^*rd7*^/J (*rd7*) (Table 1). The *rd1* mouse, representing the most severe and early form of human retinal degeneration, harbors a mutant *Pde6b* gene mapped on chromosome 5 [50–55]. The mutant *Pde6b* gene contains a murine leukemia provirus insertion in intron 1 and a point mutation, which introduces a stop codon in exon 7 (Y347STOP) [56, 57]. Independent of this, a second mutation has been found in this gene, which is the integration of a murine leukemia virus in the first intron of the 6beta (*Pde6ß*) gene [58]. Mutations in human *PDE6ß* are associated with RP and autosomal dominant congenital stationary night blindness in humans [59–61].

**Table 1:** Rate of disease progression in RP models.

Gene	Disease	Mouse model	Early/intermediate to end stage of photoreceptor degeneration	Disease progression	References
Rho	RP	*Rho*^−/−^	P30–P90	ONL degeneration begins at P30, with complete photoreceptor loss by P90	[62]
Rho	RP-4	*RhoP23H*	P15–P60	Apparent degeneration can be seen at P14, with a complete ONL degeneration by P60	[63, 107]
Cep290	LCA	*rd16*	P21–P80	ONL degeneration is observed at P21, with pigment patches seen at P14 and vessels at P30	[66]
Pde6β	Retinitis pigmentosa-40, Congenital stationary night blindness	*rd1*	P21–P50	Complete vision loss and ONL degenerated by P21	[51–55]
Nr2e3	ESCS, GFS, CPRD, and adRP	*Nr2e3*^*rd7/rd7*^	P30–P480	Whorls and rosettes present at P10, with complete ONL degeneration after P480	[69]

The rhodopsin null (*Rho*^−/−^) and the dominant negative *Rho*^*P23H*^ alleles both lack a functional rhodopsin gene [62, 63]. *Rho*^−/−^ mice lack expression of rhodopsin mRNA and protein [64]. In contrast, *Rho*^*P23H*^ mice are functional nulls with an amino acid substitution of proline to histidine at position 23 that generate an aberrant message leading to protein misfolding and degradation [63]. Specifically, RhoP23H protein undergoes incomplete glycosylation and is retained in the endoplasmic reticulum (ER) and/or Golgi apparatus where it is degraded [63]. Mutations in the human rhodopsin gene account for the largest portion of inherited retinal degenerations of known genetic etiology [65]. Further, the *Rho*^*P23H*^ mutation in particular is one of the most commonly known causes of adRP in humans [63].

The *Cep290*^*rd16*^ (*rd16*) mouse harbors a mutation in the centrosomal protein *Cep290* that results in early-onset retinal degeneration with autosomal recessive inheritance [66]. Mutations in human *CEP290* are associated with several syndromic and nonsyndromic forms of retinal degeneration [66, 67]. The *rd7* mouse is a model for *Nr2e3* associated retinal degenerations. *rd7* mice, harboring a recessive mutation in *Nr2e3*, are clinically characterized by pan retinal spots apparent at eye opening (postnatal (P) day 14), and whorls and rosettes in the outer nuclear layer (ONL) observed histologically by P10 [68, 69]. *rd7* mice have two distinct outcomes: a disruption in development of cone cells causing a significant increase of blue opsin expressing cone cells, and progressive degeneration of rod and cone photoreceptor cells [68, 70]. Results of this study show that the administration of AAV8-*Nr2e3* therapy improves clinical, histological, functional, and molecular disease outcomes in each of the five models of retinal disease. These studies demonstrate the mechanism of *Nr2e3* therapy involves resetting key retinal transcription factors and key biological networks that work in concert with *Nr2e3* to modulate the homeostatic state of the retina. This research is predicated on the fact that disease outcome is rarely due to a single gene mutation; rather, it is a result of the combinatorial mutational load on the biological system, which is often strongly influenced by other factors such as modifier genes. This study demonstrates a novel approach to gene therapy and suggests that *Nr2e3* can potentially serve as a broad-spectrum gene therapy to attenuate retinal degeneration.

## Materials and methods

### Power calculations to determine statistical significance and inclusion/exclusion criteria

Power calculations were conducted using G*Power 3.1 software analysis for estimating sample size required for each analysis and quantification described as indicated. Using means and standard deviation defined by previously published studies, a minimal number of seven animals per experimental group were used to provide 90% power and 30% difference at significant level of 0.05. The data have been analyzed double-blinded using two-way ANOVA comparisons. Animals with unresolved surgical trauma, premature unintended death, or cataracts were excluded from the study. Approximately 20% of the 600 total experimental animals were excluded from all analyses based on these criteria.

### Animal maintenance

Animals used in this study were housed and bred in the vivarium at the Schepens Eye Research Institute under standard conditions. C57BL6/J (Jax stock #000664), FVB-*Pde6ß* ^*rd1*^/NJ (*rd1*; Jax stock #001800), B6.129S6(Cg)-*Rho*^*tm1.1Kpal*^/J (*Rho*^*P23H*^; Jax stock #017628), BXD24/TyJ-*Cep290*^*rd16*^/J (*rd16*; Jax stock #000031), and *Nr2e3*^*rd7*^/J(*rd7*; Jax stock #002139) mice were obtained from Jackson Laboratories, Bar Harbor, ME. Rhodopsin Knock-out (*Rho*^*−/−*^) mice lacking expression of rhodopsin [59] were generously donated by Dr. C. Cepko (Harvard Medical School).

### AAV8-*Nr2e3* cloning and preparation

AAV8-*Nr2e3* vector was generated at the Gene Transfer Vector Core, Grousbeck Gene Therapy Center, Mass Eye and Ear (http://vector.meei.harvard.edu/). Briefly, HEK293 cells were transfected with the AAV8 rep-cap packaging, Ad-helper, and AAV2 ITR-flanked transgene constructs. After 3 days, cells and media were harvested in high salt conditions, treated with Benzonase, and cellular debris was precipitated. The supernatant was subjected to tangential flow filtration and retentate was subsequently subjected to Iodixanol ultracentrifugation density gradient. AAV fractions were collected and buffer exchange was performed for final formulation in phosphate buffered solution (PBS) + 5% glycerol. The ubiquitous CAG promoter was used in the vector. CAG is a strong synthetic hybrid promoter consisting of the cytomegalovirus enhancer fused to the chicken beta-actin promoter. Mouse *Nr2e3* cDNA to be packaged into AAV8 was generated by RT-PCR from mRNA of a B6 mouse retina using the following primers: forward: GCTGTACAAGGGCGGATGAGCTCTACAGTGGCT; reverse: ATACCGGTTGGCACTCCCAACTAGTT. These primers were introduced at the restriction sites BsrGI at the 5′ end and AgeI at the 3′ end of *Nr2e3* cDNA, and were used for cloning into the pZac2.1-CASI-eGFP-RGB plasmid (also known as pAAV). Final products were verified by restriction enzyme digestions and sequencing.

### AAV5-*Nr2e3-*GFP and AAV2.7m8-*Nr2e3* cloning and preparation

The AAV2.7m8 plasmid was obtained from Addgene, a gift from John Flannery and David Schaffer [71] (Addgene plasmid #64839: http://n2t.net/addgene:64839;RRID:Addgene_64839) (Supplementary Fig. 1). AAV5-*Nr2e3*-GFP and AAV2.7m8-*Nr2e3* were cloned and constructed by VectorBioLabs (Malvern, PA, USA), similar to the method described above for AAV8-*Nr2e3*. Mouse *Nr2e3* was introduced into restriction sites NheI and KpnI of AAV5 and restriction sites EcoRI and XhoI of AAV2.7m8 using the following primers:

Forward: CCTAAGCTTATGAGCTCTACAGTGGCTGCCTCC

Reverse: ATCGAATTCGGATCCGGTACCCTAGTTTTTGAACATGTCACACAG

The final product was verified by restriction enzyme digest and sequencing.

### Subretinal injection

All AAV-*Nr2e3* constructs were delivered by subretinal injection. Control injections included no injection in the contralateral eye, untreated animals, and GFP only injections. Approximately 600 experimental animals were used in this study. No gender bias was observed and both males (48.94%) and females (51.06%) were used equally in the study. P0 pups were anesthetized on ice, and the eyelids were carefully opened along the eyelid fissure using a 30 gauge (G) needle. The 30G needle was then used to create a hole in the sclera adjacent to the limbus, and a blunt 33G cannula attached to a Hamilton syringe was advanced into the eye. A slight resistance to the needle indicated Bruch’s membrane was reached. A total of 1 × 10^9^ viral genomes (vg) in a total volume of 0.5 μL was manually injected slowly and gently into the subretinal space of the adult or P0 mice. Subretinal injection was performed in adults as described above after anesthetizing animals by intraperitoneal (IP) injection with a mixture of ketamine (1 mg/mL) and xylazine (0.4 mg/mL).

### Clinical examination

Fundus examination and optical coherence tomography (OCT) were performed on adult injected and uninjected animals. Animals were anesthetized with a mixture of ketamine (1 mg/mL) and xylazine (0.4 mg/mL) and pupils were dilated with 1% tropicamide. Fundus images were taken using the Micron III Retinal Imaging Camera and Stream Pix software (Phoenix Research Laboratories, Pleasanton, CA, USA). Following fundus imaging, OCT was performed using the Bioptigen OCT scanner and software. Mice were restrained in a mounting tube and the fundus camera in the optical head of the apparatus and alignment was guided by monitoring and optimizing the real time OCT image of the retina. Four rotational cross section scans (dorsal–ventral and nasal–caudal) with 100 series/scan were taken for each retina. Data was analyzed using Bioptigen OCT software (*N* = 10/strain/experimental group).

### Electroretinography

Electroretinography (ERG) analysis was performed on *Nr2e3* treated and untreated animals as described previously [72]. Briefly, mice were anesthetized with an IP injection of 1 mg/mL ketamine and 0.4 mg/mL xylazine in a saline carrier (10 mg/g of body weight), and mouse eyes were dilated with 1% tropicamide and 2.5% phenylephrine hydrochloride applied topically. Dark- and light-adapted ERGs were performed using the Espion Visual Electrophysiology System (Diagnosys, Littleton, MA) with gold loop electrodes (Diagnosys LLC) placed on the apex of the cornea. A reference needle electrode was inserted subcutaneously in the forehead and a ground electrode was placed subcutaneously at the base of the tail. For scotopic recordings, mice were dark adapted for at least 6 h and then anesthetized before recording. Dark-adapted responses were recorded to short wavelength (*λ*_max_  = 470 nm; Wratten 47A filter; Kodak, Rochester, NY) flashes of light over a 4.0-log unit range of intensities (0.3-log unit steps). Light-adapted responses were obtained with white flashes (0.3 log unit steps) on a rod-saturating background after 10 min of exposure to the background light to allow complete light adaptation. Signal processing was performed using EM for Windows v7.1.2. Signals were sampled every 0.8 ms over a response window of 200 ms (LKC Technologies, Inc., Gaithersburg, MD). Responses were averaged for each stimulus condition with up to 50 records for the weakest signals. Dark-adapted responses and light-adapted responses illustrated in this study were obtained using stimulator intensities of 24.1 cd s/m^2^ for scotopic responses and 25.6 cd s/m^2^ for photopic responses (*N* = 7/strain/experimental group).

### Histology

Following euthanasia, eyes were cauterized to mark dorsal orientation, and enucleated. Tissue samples were collected and immediately immersed in freshly made 4% paraformaldehyde in 1× PBS or in 3:1 methanol/acetic acid overnight at 4 °C. Eyes were then paraffin embedded with dorsal/ventral orientation and 5 µm sections were collected over 100 µm of retinal depth and processed for hematoxylin/eosin staining. Briefly, retina sections were deparaffinated in xylene and ethanol washed and stained with hematoxylin and eosin Y. Slides were mounted with Permount mounting medium. Over 500 μm of sections/animal were visualized and representative images captured with the Leica DMI6000 microscope. As outer plexiform layer (OPL) collapse was observed in some *Nr2e3* treated retinas, the first five layers of cells were counted as inner nuclear layer (INL) and the rest of cell layers were considered as ONL when counting the ONL cell layer number. Quantification of percent observed rescue was determined by comparing treated to control B6 ONL. Cell counts were performed in a double-blinded manner over 100 μm retinal area (*N* = 10/strain/experimental group).

### Immunohistochemistry

Immunohistochemistry analysis was performed on 10 µm paraffin embedded serial sections from the enucleated mouse eyes as described in our previous studies [72]. At minimum 100 µm of retina/sample was evaluated by IHC. Briefly, sections were blocked with 2% normal horse serum (#S-2000 VectorLabs, CA) in PBS, and incubated with the following cell type-specific primary antibodies in a 1:200 dilution: rhodopsin (mouse monoclonal, Millipore MAB5356); green/red opsin (rabbit polyclonal, Millipore AB5405); blue opsin (rabbit polyclonal, Millipore AB5407); GFP (1:500, rabbit polyclonal, Abcam ab290). The following day, sections were rinsed with PBS and incubated with the corresponding secondary antibody (1:400 Alexa fluor 488 goat antirabbit, Invitrogen A11008) and nuclei were stained with 4,6-Diamidino-2-Phenylindole, Dihydrochloride (DAPI). Over 500 μm of sections/animal were visualized and representative images of IHC labeling were captured using a Leica DMI6000 fluorescent microscope equipped with the appropriate bandpass filter for each fluorochrome. Cell counts were performed in a double-blinded manner over 100 μm retinal area (*N* = 10/strain/experimental group).

### Retinal whole mount IHC

Retina whole mounts have been performed as previously described [73]. Microdissection of the retina for whole mounts were performed as follows: the anterior eye segments, including the iris, were removed with a round cut along the limbus using microdissection scissors. The lens was then removed and the retina was gently separated from the pigmented epithelium and the choroidal-scleral complex. Whole retinas were transferred to a 96-well culture plate and immunohistochemistry was performed as follows. Retina cups were blocked with 2% normal horse serum (#S-2000 VectorLabs, CA) in PBS with 0.02% sodium azide, 1% bovine serum albumin (BSA), and 0.1% Triton X 100; and then incubated with the following cell type-specific primary antibodies in a 1:100 dilution: rhodopsin (mouse monoclonal, Millipore MAB5356), green opsin (rabbit polyclonal, Millipore AB5405) and blue opsin (rabbit polyclonal, Millipore AB5407) overnight, and incubated with the corresponding secondary antibody (1:200 Alexa fluor, Invitrogen) overnight. Retinas were flowered with radial incisions: two horizontal and two vertical incisions at 3, 6, 9, and 12 clock, starting from the edge toward the optic nerve and cutting 2/3 of the distance from the periphery to the center, giving the retina a cross-like form. Retinas were flat mounted on a microscope slide. Opsin labeling was visualized and images captured using a Leica DMI6000 fluorescent microscope equipped with the appropriate bandpass filter for each fluorochrome (*N* = 10/strain/experimental group).

### Quantitative real time PCR (qRT-PCR)

Total RNA was extracted from whole retinas using the Trizol method as described previously [72]. Briefly, 2 µg of total RNA was reverse transcribed using Retroscript (Ambion AM1710) to generate cDNA. The cDNA samples were diluted 1:100 and real time PCR was performed in triplicates for each primer using Sybr Green PCR master mix (Thermo fisher #4309155). The real time PCR primers (Supplementary Tables 1 and 2) were designed using NCBI Primer-Blast and were specific for each target gene. Reactions were quantified using an ABI Step One Plus Real Time PCR and analyzed with the corresponding software. Relative expression levels were determined by normalizing cycle threshold values to the amount of β-actin expressed (1000/2^Ct^ gene—Ct β-actin). Statistical significance of differential expression was assessed using a *T*-test and *P* value of <0.05. Results are mean ± SEM (*N* = 7). Primers amplifying SV40 from AAV8 vector were used to determine exogenous *Nr2e3* expression (F:AGCAATAGCATCACAAATTTCACAA; R:CCAGACATGATAAGATACATTGA).

### Chromatin immunoprecipitation—RT-PCR

Chromatin immunoprecipitation (chIP) was performed using P30 C57Bl6/J mouse retinas as previously described [49]. A total of 8–10 retinas were used per chIP reaction. Briefly, tissue was dissociated, homogenized, and cross-linked in 37% formaldehyde and sonicated to generate sheared fragments of 400–600 bp. Immunoprecipitation was performed overnight using 1 μg of *NR2E3* antibody, goat IgG antibody served as a negative control, and the input (positive control) was not incubated with antibody. Immunoprecipitated samples were reverse cross-linked. *Nr2e3* putative target genes were analyzed for nuclear receptor response element (RE) binding site using the classic (AAGTCA (*n* = 1–4) AAGTCA) RE binding sequence of *Nr2e3* as determined algorithmically by NUBIscan124. Binding sites were searched for in a maximum of 100 kb upstream region of each gene’s start site and into intron 1. Real time primers were selected flanking putative RE sites with an average amplicon size of 200 bp (Supplementary Table 3). Quantitative RT-PCR was performed using 1 µl of 1:100 dilution (input) and 1:10 dilution (samples and immunoglobulin G (IgG) control). All sample data were normalized to IgG control. Results are mean ± SEM (*N* = 7/strain/experimental group).

## Results

### Overexpression of *Nr2e3* has no detrimental effect on the retina

**Fig. 1:**
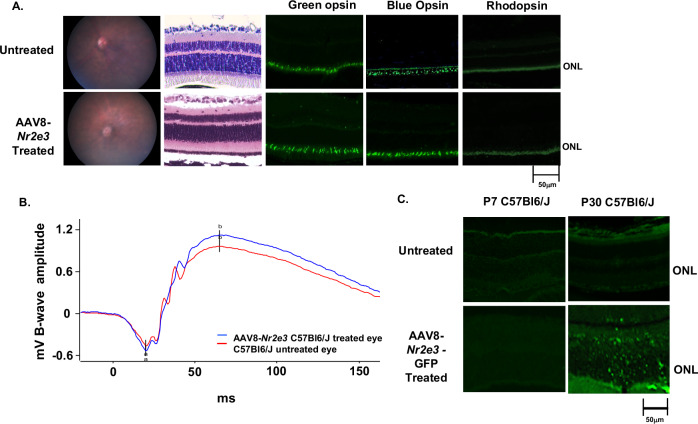
Overexpression of AAV8*-Nr2e3* has no detrimental effects on the retina. B6 control AAV8-*Nr2e3* treated animals show no abnormalities in **a**. Fundus, hematoxylin/eosin histology staining, and blue, green, and rhodopsin labeling of photoreceptor cells; and **b** ERG response of control B6 treated and untreated. Animals injected at P0, tissue collected at P30. **c** GFP label of AAV8*-Nr2e3-GFP* injected at P0, GFP expression assessed at P7 and P30. *N* = 5.

C57BL6/J (B6) animals were treated with AAV8-*Nr2e3*-GFP fusion protein to evaluate any potential detrimental effects of overexpression of *Nr2e3*, as well as the timing of construct expression post delivery. B6 mice were injected at P0 and evaluated at P7 and 1 month. No observable degeneration was detected in the retina post injection (Fig. 1a). Consistent with clinical findings, AAV delivery of *Nr2e3* did not cause aberrant morphological changes, and immunolabeling of rod and cone opsins revealed no observable difference between injected and uninjected animals (Fig. 1a). Functional output of the retina, as detected by electroretinogram (ERG) of rod and cone responses, showed no significant differences between injected and uninjected eyes (Fig. 1b). Examination of AAV8-EGFP*-Nr2e3* expression at P7 and P30 revealed that expression of the vector construct was confirmed at P30 (Fig. 1c). These results confirm that overexpression of *Nr2e3* by subretinal AAV8-*Nr2e3* injection is not detrimental to the retina.

### Vector or GFP alone do not affect the retina

**Fig. 2:**
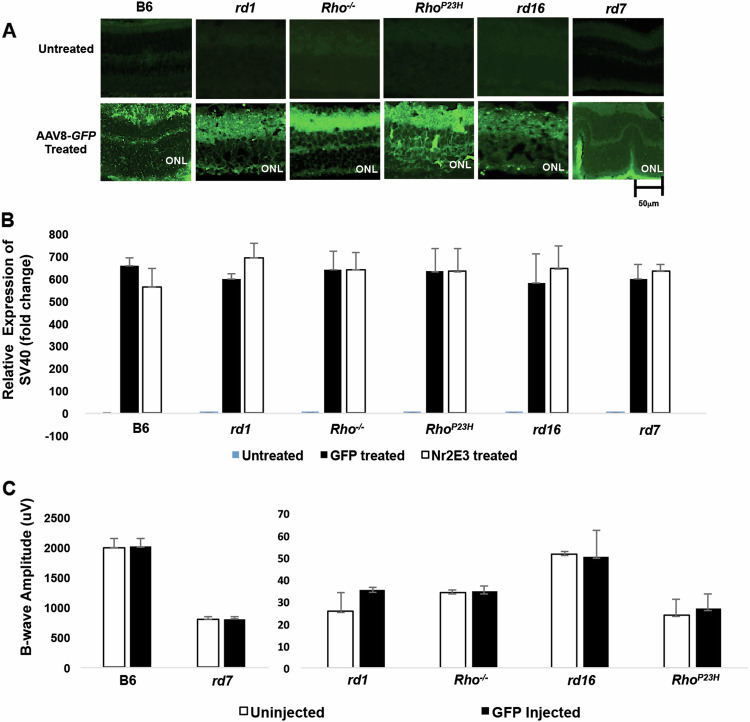
Expression of AAV8-GFP at P30 in RP models has no effect. **a** Immunohistochemistry of AAV8-GFP (*rd1, Rho*^−/−^*, Rho*^*P23H*^*, rd16*, and *rd7*). All RP models except *rd7* have only 0–1 cells in the ONL at P30 and GFP expression is more pronounced in other layers yet has no impact on disease. **b** Semiquantitative analysis of SV40 (part of AAV8) expression in untreated, AAV8-GFP, and AAV8-*Nr2e3* retinas of B6 control and RP models relative to beta-actin. **c** ERG B-wave amplitudes of uninjected and AAV8-GFP injected RP models and B6 control. Animals injected at P0, tissue evaluated at P30. ERGs recorded at P30. Results are mean ± SEM. *N* = 7.

Animals were injected with AAV8-*EGFP* at P0 and evaluated at 1 month to demonstrate that an empty vector alone is not sufficient for or contributes to the rescue observed in *Nr2e3* treated animals. Immunohistochemistry analysis confirmed vector expression without any abnormal morphological changes (Fig. 2a). All models except *rd7* have 0–1 cells in the ONL by P30, thus GFP is observed in other layers yet has no impact on the disease. Semiquantitative analysis of SV40 polyA gene expression shows no significance difference in expression of SV40 in AAV8-*EGFP* treated retinas as compared with AAV8-*EGFP-Nr2e3* treated animals (Fig. 2b). ERG analysis also suggests that there is neither rescue nor any detrimental effect on functionality of the retina of all mutant strains when treated with empty vector only (AAV8-*EGFP*) (Fig. 2c).

### AAV delivery of *Nr2e3* in RP models before disease onset attenuates retinal degeneration

**Fig. 3:**
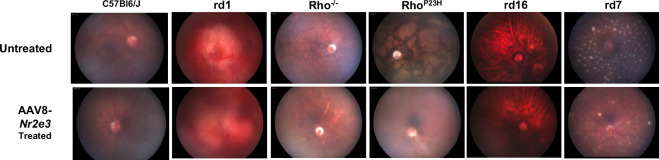
AAV8*-Nr2e3* rescues clinical phenotype in multiple mouse models of RP. Fundus of P0 injected AAV8*-Nr2e3* treated and untreated animals evaluated at P30 (B6 and *rd1*) or P90-P120 (*Rho*^−/−^*, Rho*^*P23H*^*, rd16*, and *rd7*). *N* = 7.

The ability of *Nr2e3* to rescue retinal degeneration before disease onset was tested by subretinal delivery of AAV8-*Nr2e3* in five mouse models of RP. All models except *rd1* were injected at P0 and evaluated at 3–4 months of age. *rd1* animals were injected at P0 and evaluated at 1 month of age due to their accelerated rate of disease progression. Although not all models have a clinical phenotype, considerable improvements were observed in the fundus of *Rho*^*P23H*^, *rd16*, and *rd7* mice (Fig. 3). Interestingly, we observed the *rd16* mice have a red fundus with increased and pronounced vessels (not previously reported). While no vascular leakage has been observed in *rd16* mice when examined by fluorescein angiography (our unpublished data), the fundus observation resolves with *Nr2e3* administration. Improvement was observed in the *rd7* phenotype, with reduction of retinal spots in AAV8-*Nr2e3* treated eyes compared with untreated eyes at 3 months post injection.

**Fig. 4:**
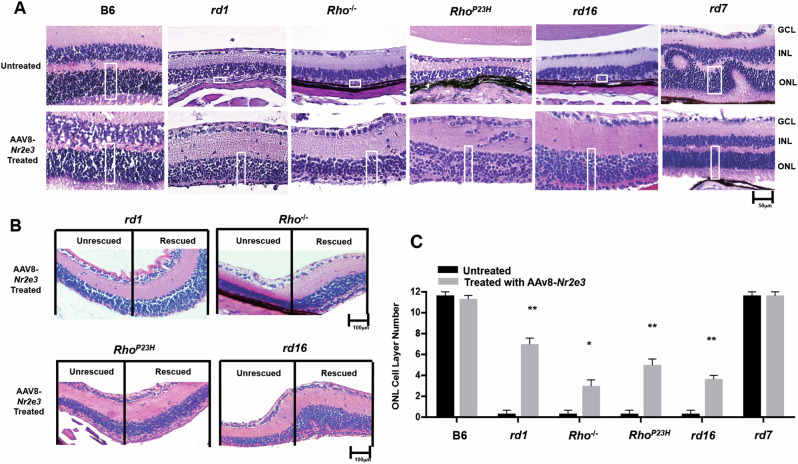
AAV8-*Nr2e3* treatment preserves retinal morphology and retinal integrity in RP models. B6, *rd1, Rho*^−/−^*, Rho*^*P23H*^*, rd16*, and *rd7* animals injected at P0, evaluated at P30 (B6 and *rd1*) or P90-P120 (*Rho*^−/−^*, Rho*^*P23H*^*, rd16,* and *rd7*). **a** Hematoxylin/eosin staining of AAV8-*Nr2e3* treated and untreated retinas with white boxes indicating location of cell count. **b** Rescued and un-rescued regions in retinas treated with AAV8*-Nr2e3*. **c** Cell layer numbers of ONL from AAV8*-Nr2e3* treated and untreated animals in different RP models. Results are mean ± SEM. *N* = 7.

Photoreceptor degeneration often disrupts retinal topography and present with abnormal morphology. Histology analysis shows AAV8-*Nr2e3* therapy improves retinal morphology and integrity in RP models. The normal mouse retina is comprised of 10–12 layers of rod and cone photoreceptor nuclei in the ONL and 5–6 layers of inner retinal cells in the INL. In the retinal degeneration mouse models evaluated, the INL did not change in nuclei number, and the ONL presented zero or only one layer of cells at the time of evaluation, with the exception of the *rd7* model that presents with increased cone cells with whorls and rosettes in the ONL. Hematoxylin/eosin (H/E) staining revealed that subretinal delivery of AAV8-*Nr2e3* at P0 rescued photoreceptor cells and helped maintain retinal integrity of RP retinas in all models tested at 1 month (*rd1*) post treatment, or 3–4 months (*Rho*^−/−^, *Rho*^*P23H*^, *rd16*, and *rd7*) post treatment (Fig. 4a).The attenuation of disease phenotype, as observed by ONL thickness, varied among each strain. Partial rescue (~30–80%) of the ONL count in all treated retinas was observed (Fig. 4b). It is also noteworthy that there is absolutely no gender bias in the rescue of animals as 50% of the rescued animals are male and 50% are female. Interestingly, retinal whorls and rosettes that are characteristic of the *rd7* phenotype, resolved following *Nr2e3* treatment, suggesting that the delivery of *Nr2e3* at P0 can restore normal retinal development (Fig. 4a). Although a clear boundary between INL, ONL, and OPL was difficult to visualize in some *Nr2e3* treated retinas, ONL was significantly increased in the rescued portion of treated retinas (Fig. 4b). *rd1* retinas showed a profound rescue of photoreceptor cells, with a total of 6–8 nuclei layers observed in the treated eye. *Rho*^−/−^*, RhoP23H*, and *rd16* mice showed a more moderate increase of 3–6 layers of ONL in *Nr2e3* treated retinas compared with 0–1 layer in the untreated eyes of each model (Fig. 4c). Although only partial rescue was observed in all models, studies in patients have demonstrated that retention of only a single layer of photoreceptor cells can be enough to maintain minimal visual function suggesting that an increase of even 20% is significant [61, 74]. Thus, *Nr2e3* therapy could have great promise in restoring retinal development.

### AAV8*-Nr2e3* therapy preserves cone and rod opsin expression in five models of RP

**Fig. 5:**
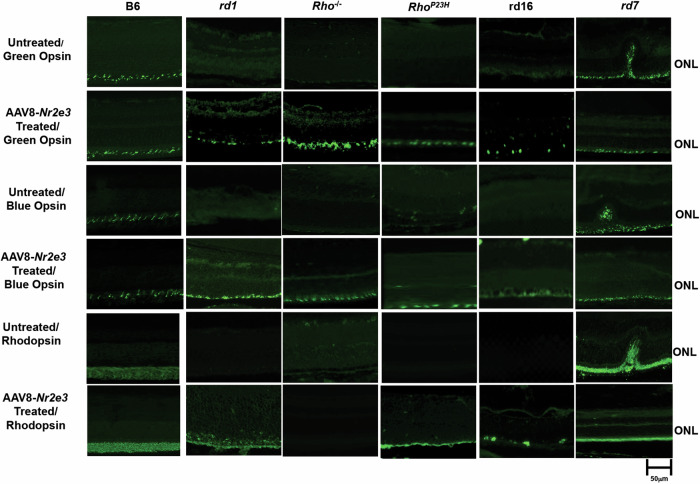
AAV8-*Nr2e3* preserves cone and rod opsin expression in multiple mouse models of RP. Immunohistochemistry of P0 injected AAV8-*Nr2e3* treated and untreated retinas labeled with green opsin, blue opsin and rhodopsin evaluated at P30 (*rd1*) or P90-P120 (*Rho*^−/−^*, RhoP23H, rd16*, and *rd7*) and B6 control. *N* = 7.

**Fig. 6:**
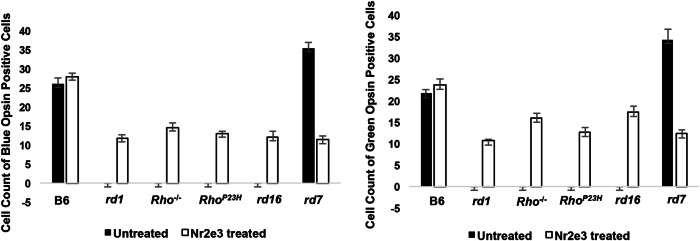
Cell counts of blue and green opsin confirm preservation following AAV8-*Nr2e3*. Semiquantitative analysis of cell counts of blue and green opsin-positive photoreceptor cells per 100 μm. Results are mean ± SEM. *N* = 7.

**Fig. 7:**
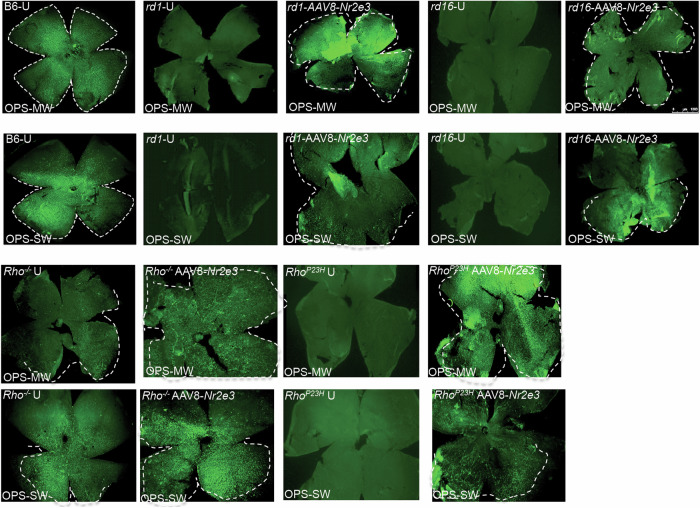
Cone Opsin topography improved in AAV8-*Nr2e3* RP retinas. Whole mounts of green opsin and blue opsin were evaluated at 1-month old C57Bl6/J control, as well as 1-month *rd1*, *Rho*^−/−^, *Rho*^P23H^, and *rd16* animals treated with AAV8*-Nr2e3* at P0 and untreated animals. *N* = 7.

Immunohistochemical analysis of blue and green cone opsins and rhodopsin was performed to determine if *Nr2e3* therapy can restore opsin expression and thus provide a molecular reset in retinal degeneration models. Eyes from treated and untreated animals were collected at 1 month (*rd1*) or 3–4 months (*Rho*^−/−^, *Rho*^*P23H*^, *rd16*, and *rd7*) post injection and labeled with antibodies to green and blue cone opsins and to rhodopsin for rods. Untreated eyes showed no opsin-positive photoreceptor cells, except that of *rd7* and *Rho*^*−/−*^. Consistent with our previous publication, *rd7* retinas show an increase in blue opsin and slow progressive loss of all opsins over 5–16 months (Fig. 5) [70]. These studies show *Rho*^−/−^ mice have sparse expression of blue and green opsin expressing cones at 1 month (Fig. 5). Interestingly, rhodopsin expression was observed in *Rho*^*P23H*^ retinas treated with *Nr2e3*. The semiquantitative analysis of the blue and green opsin-positive cells shows that there is partial rescue of photoreceptor cells in *rd1*, *Rho*^−/−^, *Rho*^*P23H*^, and *rd16* (Fig. 6). En face view of blue and green opsin expression in whole mount retinas of *rd1*, *Rho*^*−/−*^, *Rho*^*P23H*^, and *rd16 Nr2e3* treated animals confirm observations of retinal sections with partial rescue of cone opsin expression in each model, consistent with H/E stain of partial rescue of ONL cells (Fig. 7, opsin expressed regions outlined by a dashed line). By 1-month age, no expression of blue or green opsin was observed in untreated *rd1*, *Rho*^*P23H*^, and *rd16* animals. Similar to IHC in sections, *Rho*^−/−^ retinas show sparse expression of blue and green opsin. In contrast, all treated animals showed restored blue and green opsin expression (Fig. 7). IHC performed on 1 or 3 month treated animals show consistent expression of the cone opsin genes demonstrating sustained rescue.

### Improved ERG responses observed in AAV-*Nr2e3* treated RP retinas

**Fig. 8:**
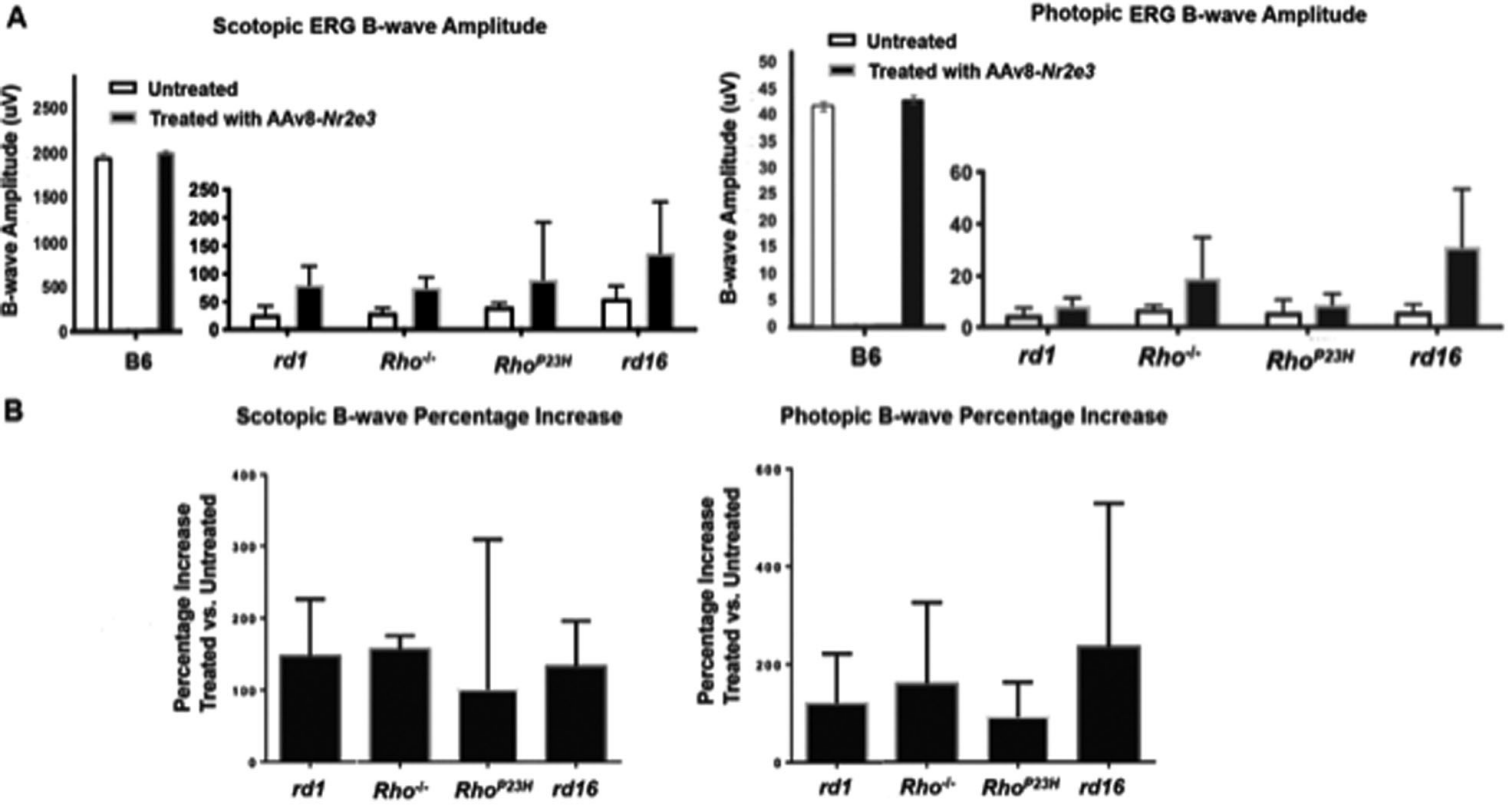
Improved ERG responses in AAV8-*Nr2e3* treated RP retinas. **a** Scotopic and photopic ERG B-wave amplitudes were evaluated at P30 (*rd1*) or P90-P120 (*Rho*^−/−^, *Rho*^P23H^, and *rd16*) AAV8-*Nr2e3* treated and untreated animals; B6 control ERGs shown. **b** Percent increase in ERG B-wave responses in the treated RP models. Results are mean ± SEM. *N* = 7.

RP disease progression results in the loss of rod and cone function that is assessed by abnormal ERG responses [62, 63, 75–79]. Our previously published study showed improvement of ERG in *rd7* mice with *Nr2e3* [70]. In the present study, the visual function of *Nr2e3* treated RP retinas was examined in four out of five RP strains, excluding *rd7*, by recording dark-adapted and light-adapted ERGs to evaluate rod- and cone-driven responses. Consistent with histology and IHC studies, partial rescue is observed in *Nr2e3* treated animals compared with untreated animals (Fig. 8a). Significant percent increase of the scotopic amplitudes is observed in the treated mutant mice compared with untreated controls in each model. (Fig. 8b).

### AAV8-*Nr2e3* preserves retinal homeostasis in RP retinas

**Fig. 9:**
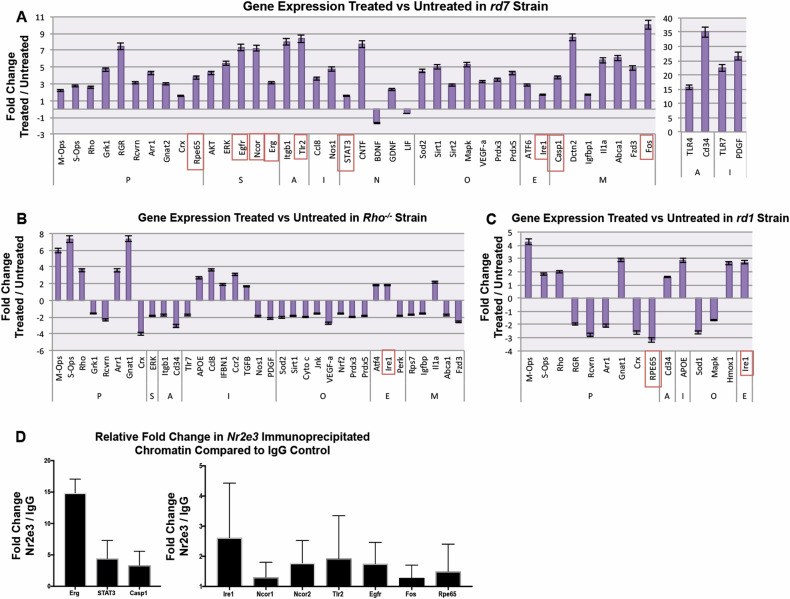
AAV8-*Nr2e3* treated retinas exhibit differential expression in multiple key homeostasis gene networks. **a**–**c** Differentially expressed genes from *Nr2e3*-directed networks with ≥1.5-fold variance in *Nr2e3* treated vs untreated retinas in *rd7, rd1*, and *Rho*^−/−^, respectively. Networks P phototransduction, S survival, A apoptosis, I immunity, N neuroprotection, O oxidative stress, E ER stress, M metabolic. Potential target genes of *Nr2e3* are highlighted. **d** Potential *Nr2e3* targets by Chromatin IP—real time PCR. P0 injected, samples collected at 3 months. Results are mean ± SEM. *N* = 7.

NHR genes such as *Nr2e3* play key roles in modulating homeostasis by regulating many key biological processes and gene networks. Our prior studies and recent Ingenuity Pathway Analysis revealed that *Nr2e3* regulates several key biological networks that are critical to maintaining retina homeostasis in the retina including phototransduction, cell survival, apoptosis, immunity, oxidative stress, ER stress, neuroprotection, and metabolism [49]. Representative subsets of treated animals (*rd7, Rho*^−/−^, and *rd1*) were evaluated for differential expression of genes that function in *Nr2e3* regulated pathways. Seventy-five genes were evaluated from eight *Nr2e3* modulated biological networks for *Nr2e3* RE binding sites. We identified putative *Nr2e3* binding sites in 19 out of 75 genes. Genes with a ≥1.5-fold variance change between the AAV8-*Nr2e3* treated and untreated eyes were considered statistically significant. Consistent with the IHC results, the representative strains exhibit a significant change in gene expression of the opsin genes (Fig. 9a–c). Each strain had a unique set of genes that were differentially expressed between treated and untreated animals. Five out of eight networks were modulated by *Nr2e3* treatment in each strain. As expected, the *rd7* treated retinas had the greatest number of genes with differential expression in treated vs. untreated retinas (Fig. 9a). In addition, 10 genes were identified by chromatin immunoprecipitation (chIP)-RT-PCR as potential direct targets of *Nr2e3* (Fig. 9d), nine of which were differentially expressed in *rd7* treated retinas. Interestingly, the ER stress and cell survival factor, inositol—requiring enzyme 1 (*Ire1*)—is a potential direct target of *Nr2e3* and is differentially expressed in all treated animals (Fig. 9a–c). Considering the unique mutational load of each model, it is not surprising that each mutant had a unique consortia of genes and networks reset by *Nr2e3* treatment. Consistent with previous work demonstrating that *Nr2e3* is a dual activator/repressor [39, 46–49, 80, 81], these genes were differentially modulated. These results illustrate that mutational load is modulated and balanced by transcription factors, including NHRs, for optimal cellular homeostasis [82, 83]. The upregulation of *Ire1* in all treated models suggests at least one common network (ER stress and the promotion of cell survival) through which *Nr2e3* modulates and resets homeostasis. Collectively, these findings show that while the specific reset varies among diseases, administration of *Nr2e3* to RP diseased retinas has a positive impact in restoring the homeostatic state of the retina in the presence of disease, thus attenuating disease progression.

Studies completed by our lab and others have extensively documented the importance of *Nr2e3* in photoreceptor development [69, 80, 81, 84]; however, the role of *Nr2e3* in the mature retina is less understood. Recent studies reveal a key regulatory role for *Nr2e3* in maintaining proper function of mature photoreceptor cells [69, 80].

**Fig. 10:**
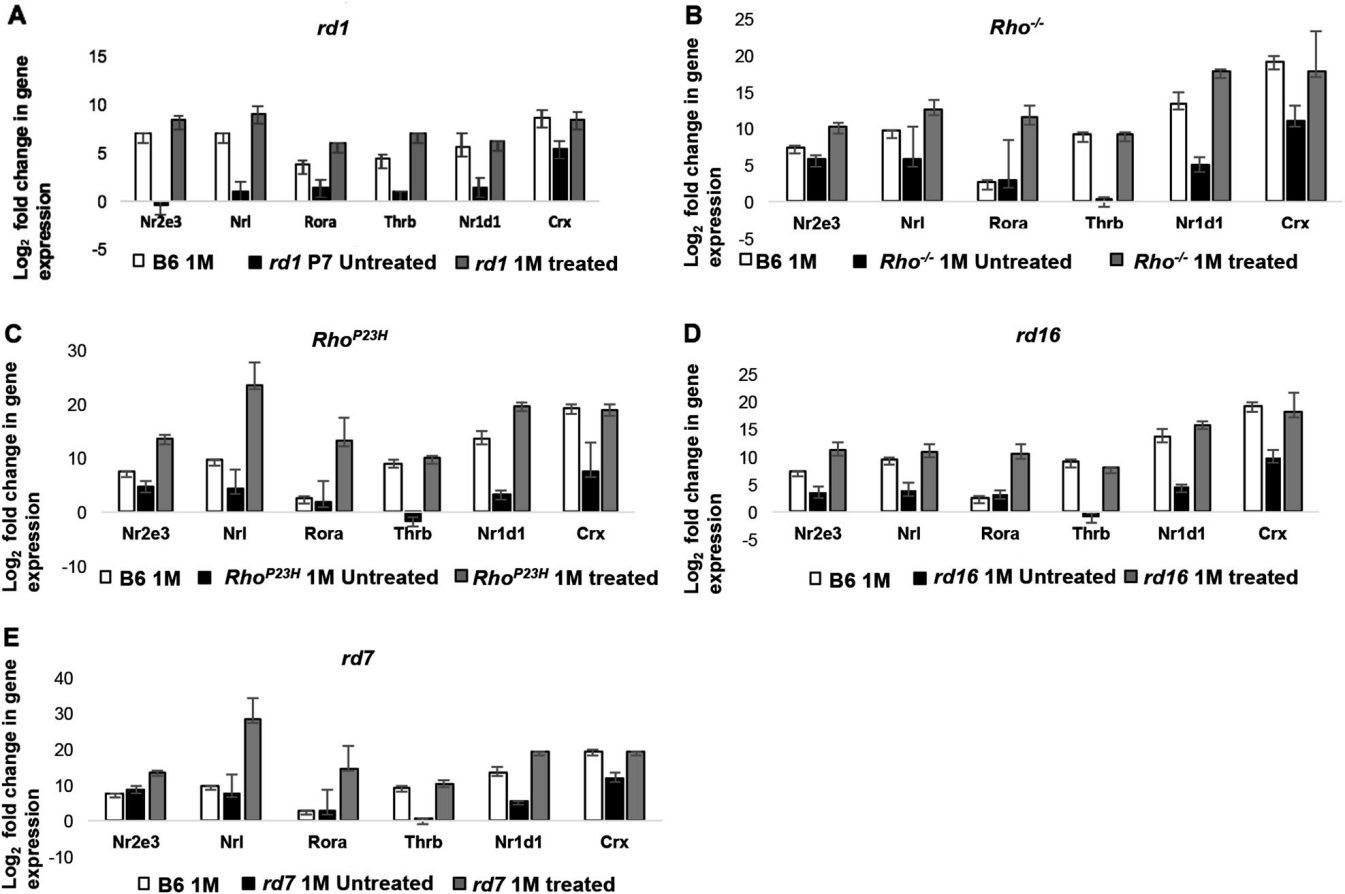
AAV8-*Nr2e3* rescues RP degeneration by recruiting key transcription factors. Relative expression levels of *Nr2e3*, *Nrl*, *Rora*, *Thrb*, *Nr1d1*, and *Crx* at P30 *Nr2e3* treated mutant strains (*rd7, Rho*^−/−^*, Rho*^P23H^, and *rd16*) and *rd1* at P7 compared with the corresponding untreated controls and normalized to beta-actin. Results are mean ± SEM. *N* = 7.

The expression of *Nr2e3* in all five RP mutant models was evaluated to determine if the loss of *Nr2e3* contributes to RP disease. Interestingly, *Nr2e3* expression in P7 (*rd1*) and P30 RP retinas showed a significant decrease all RP models except in the *rd7*^−/−^ model. Our previous studies showed that the *rd7*^−/−^ mouse, a functional null of *Nr2e3*, has high *Nr2e3* mRNA expression but lacks protein expression [48]. These results suggest that the loss of *Nr2e3* expression likely contributes to the retinal degeneration observed in each model, and the addition of *Nr2e3* provides a reset for the for transcriptional signature of treated retinas (Fig. 10). *Nr2e3* has been shown to function with other transcription factors such as *Nr1d1*, neural retinal leucine zipper (*Nrl*), Cone-rod homeobox (*Crx*), retinoic acid receptor related orphan receptor alpha (*Rora*), and thyroid receptor beta (*Thrb*) to modulate photoreceptor cell fate and retinal function as an activator or suppressor of gene expression [47–49, 80, 81, 85–87]. The expression level of five other essential retinal transcription factors (*Nr1d1, Nrl, Crx, Rora*, and *Thrb*) were determined in *Nr2e3* treated and untreated retinas. Overall, a significant decrease in expression of key retinal transcription factors was reversed following *Nr2e3* therapy (Fig. 10). *rd1* mice lacked expression of all transcription factors tested except *Crx*, and these were restored with *Nr2e3* therapy, significant down regulation (Fig. 10a). Both the rhodopsin models and *rd16* exhibited an overall decrease in these transcription factors (Fig. 10b–d), and *rd7* exhibits an overall reduction of transcription factor expression that is reset following *Nr2e3* therapy (Fig. 10e).

### AAV8-*Nr2e3* rescues retinal degeneration after disease onset

**Fig. 11:**
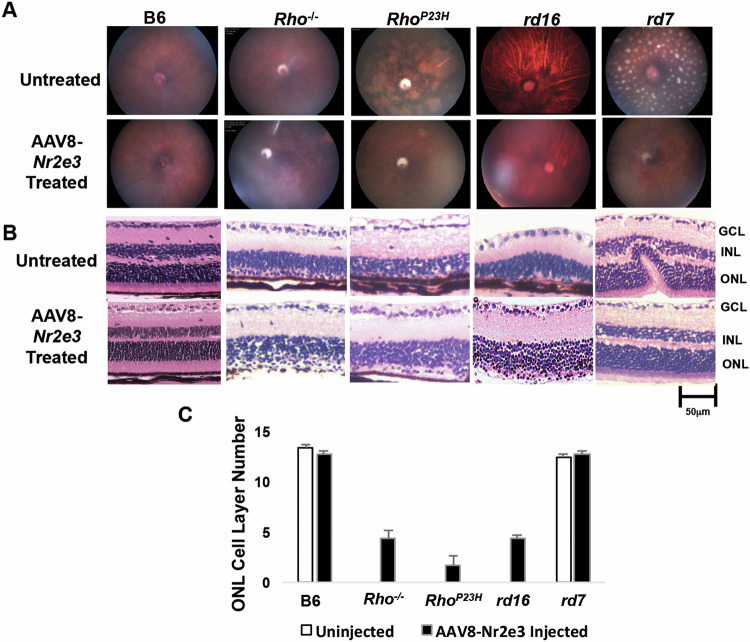
AAV8*-Nr2e3* rescues RP degeneration after disease onset. Animals injected with AAV8*-Nr2e3* at P21 and evaluated at 2–3 months post injection. **a** Fundus of *Rho*^−/−^*, Rho*^*P23H*^*, rd16*, and *rd7*. **b** Hematoxylin/eosin staining shows partial preservation of photoreceptor cells in treated mutant animals. **c** Cell layer numbers of outer nuclear layer were compared between AAV8*-Nr2e3* treated and untreated animals in the four RP models and B6 control. Results are mean ± SEM. *N* = 7.

**Fig. 12:**
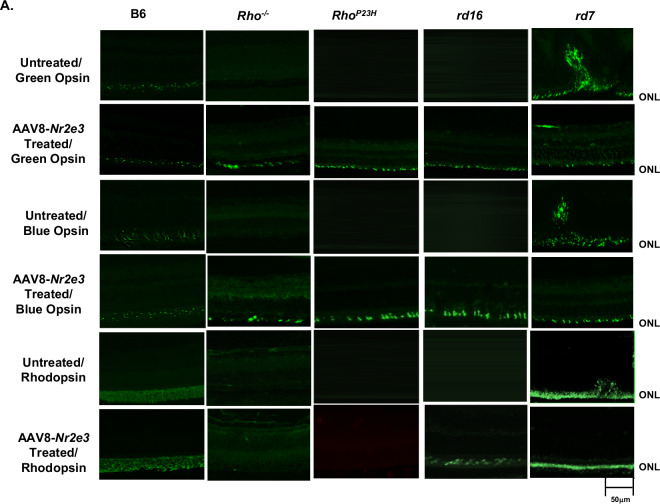
AAV8-*Nr2e3* rescues rod and cone opsin expression after disease onset. Animals were injected with AAV8-*Nr2e3* at P21 and evaluated at 2–3 months after injection. Immunohistochemistry of green opsin, blue opsin and rhodopsin of treated and untreated animals in *Rho*^−/−^*, RhoP23H, rd16*, and *rd7*.

**Fig. 13:**
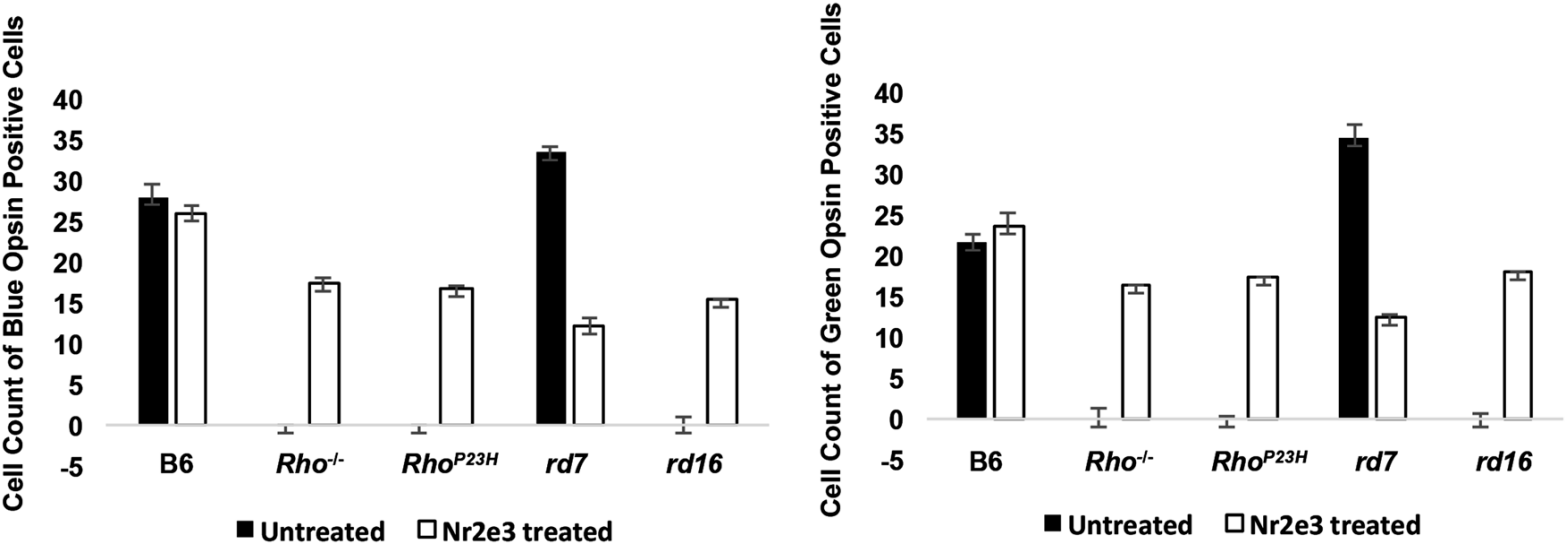
Cell counts of blue and green opsin confirm rescue following AAV8-*Nr2e3*. Semiquantitative analysis of cell counts of blue and green opsin-positive photoreceptor cells per 50 μm of the retina. Results are mean ± SEM. *N* = 7.

AAV8-*Nr2e3* was administered at early to intermediate stage of disease (Table 1) to determine the efficacy of *Nr2e3* modifier gene therapy at a time that better represents clinical presentation. AAV8-*Nr2e3* was injected subretinally at P21 and evaluated 2–3 months post injection in *Rho*^*−/−*^, *Rho*^*P23H*^, *rd16*, and *rd7* mice. *rd1* mice were injected earlier than P21 as their ONL rapidly degenerates during development. Fundus and histology show the attenuation of retinal degeneration by *Nr2e3* therapy in each model (Fig. 11a, b). As shown previously, improvement varied from ~30 to 80% of the retina, depending on distribution efficiency throughout the retina. Approximately three to five layers of ONL cells were preserved in *Nr2e3* treated animals compared with untreated animals that show less than or equal to one layer of ONL remaining (Fig. 11c). IHC labeling of blue and green cone opsins and rhodopsin further demonstrated the capability of *Nr2e3* therapy to rescue photoreceptors after disease onset (Fig. 12). The semiquantitative analysis of the blue and green opsin-positive cells shows a partial rescue of photoreceptor cells in *rd1*, *Rho*^−/−^, *Rho*^*P23H*^, and *rd16* (Fig. 13). Rhodopsin rescue is noteworthy in *Rho*^*P23H*^ mice when treated at P0 or P21 (Figs. 5, 12, and 13), emphasizing the unique capability of *Nr2e3* to modulate disease mechanism spatially and temporally.

To confirm that the rescue observed in *Nr2e3* treated retinas is not vector specific, *Nr2e3* was packaged in AAV5 and AAV2.7m8 [71, 88]. Adult *rd7* animals were injected with AAV5-*Nr2e3*-GFP or AAV2.7m8-*Nr2e3* and evaluated clinically before treatment, as well as 1 month post treatment. The presence of GFP in the AAV5-*Nr2e3*-GFP treated retinas correlated with the region of rescue and corresponded to absence of retinal spots (Supplementary Fig. 2). Reduction of retinal spots and retinal whorls was observed in *rd7* animals after 1 month of AAV5 or AAV2.7m8-delivered *Nr2e3* (Supplementary Fig. 2). OCT images were scanned and captured at the same frame for the same animal before treatment and 1 month post treatment. These results demonstrate that *Nr2e3* can ameliorate retinal degeneration outcomes regardless of the vehicle of delivery.

## Discussion

Many factors strongly influence clinical outcome of disease including genetic modifiers (such as *Nr2e3*), epigenetic factors, allelic heterogeneity, and the environment [40]. Genetic modifiers play a key role in modulating disease onset, progression, and clinical outcome by either enhancing or suppressing disease [24, 40]. The discovery of genetic modifiers has provided a novel and innovative opportunity to develop powerful therapeutic strategies to treat human diseases. While tremendous work has focused on developing novel gene replacement and gene editing strategies to target mutant genes for diseases such as RP [89–93], it is not possible or ideal for many diseases and broader approaches must be developed. This is the first study to demonstrate successful improvement in several different mouse models of retinal degeneration by a single genetic modifier gene. Each strain showed improvement of photoreceptor survival, preservation of retinal structure, a change in gene expression, and stabilization of retinal function. While the specific reset varied between species, the cumulative impact toward the restoration of a more homeostatic state of the retina was observed. This restoration allowed for sustained impact and attenuation of RP disease.

We observed 30–80% improvement at the histological, immunohistochemical, and functional level in five RP models following treatment with AAV8-*Nr2e3*. AAV8 was chosen for this study due to its ability to target specificity toward photoreceptor cells. In addition, *Nr2e3* expression was driven by a strong, general promoter rather than a cell type-specific promoter, as they are often not as robust. The models used in this study were chosen because they vary on severity and rate of disease progression, modeling human inherited retinal degeneration. Restoration of photoreceptor cells was consistently observed in each model by several methods. Histological analysis revealed regions of rescue in each treated eye. The rescue was demonstrated as an increase in ONL nuclei in specific regions of the retina. Similarly, IHC showed improvement of cone and rod opsin expression in regions of rescue, and it is easy to distinguish regions of rescue in retinal sections and en face views with whole mount retinas. While analysis of individual ERGs showed similar levels of improved responses in animals that showed clinical, histological, and molecular improvement, the signal to noise ratio prevented ERG analysis from confirming a robust cumulative effect by full-field ERG recordings in comparison to focal ERGs. Several factors such as dosage and variation of injection site can contribute to the variability observed in the degree of rescue per therapy treatment. In addition, the scotopic ERG response improvements in *Rho*^−/−^ mice likely come from cone responses, as IHC and flat mount data demonstrate exclusive rescue of cone opsins and lack of rhodopsin expression in treated and untreated.

It is noteworthy that these studies were conducted using a low dose of AAV8-*Nr2e3*, 1 × 10^9^ genome copy, which may also contribute to the modest improvement observed for some treated retinas. AAV expression has been shown to be present for months, and even years, in higher order animals, suggesting that while it is not integrated, it does provide long term stable expression [94–96]. The potency of *Nr2e3* as a therapeutic is clearly demonstrated by its ability to attenuate retinal degeneration and restore retinal integrity in several RP models at the early to intermediate stage of the disease. In addition, our prior and current studies show that regardless of the vehicle (naked DNA, nanoparticle, AAV8, AAV5, or AAV2.7m8), *Nr2e3* can rescue retinal disease in *rd7*.

Our prior studies demonstrated the role of *Nr2e3* in modulating numerous gene networks that impact homeostasis. Here, we evaluated 75 genes from eight key *Nr2e3*-directed gene networks that contribute to retinal homeostasis to determine the mechanism by which *Nr2e3* achieved such broad-spectrum rescue. Evaluating genes in 8 of the *Nr2e3*-directed gene networks revealed that improved outcome coincides with differential expression of genes in the *Nr2e3*-directed networks. These gene networks are key regulators maintaining the homeostatic state of the retina. While each mutant strain is directed by a unique transcriptional profile, common networks such as phototransduction, apoptosis, immunity, oxidative stress, and ER stress genes were impacted in each treated model

One biological regulatory network influenced by *Nr2e3* is ER stress. For example, *Ire1*, a putative direct target of *Nr2e3*, is upregulated in treated eyes of all models tested. *Ire1* encodes an ER-resident transmembrane protein that controls the most highly conserved unfolded protein response (UPR) signaling pathway during ER stress [97, 98]. *Ire1*, a potential gene target of *Nr2e3*, is upregulated in all *Nr2e3* treated eyes compared with their respective controls. The ER is an essential organelle for multiple cellular functions including maintaining calcium homeostasis and the biosynthesis of proteins and lipids. Protein misfolding and the consequential accumulation of unfolded proteins in the lumen of the ER initiate the UPR to alleviate stress [99]. *Ire1* undergoes oligomerization and activation of its kinase and RNase functions in response to these protein misfolding in the ER, initiating the nonconventional splicing of Xbp-1 mRNA [100]. Thereby, *Ire1* signaling enhances the protein-folding environment of the ER by expanding the amount of ER and its constituent protein-folding machinery, as well as clearing misfolded ER proteins [101]. *Ire1* signaling pathway transcriptionally upregulates numerous ER chaperones that were shown to promote P23H rhodopsin exit from the ER by partially ameliorating this misfolding defect [102–104]. The *Rho*^*P23H*^ point mutation encodes a misfolded rhodopsin protein, one which undergoes incomplete glycosylation and is retained in the ER and/or Golgi where most of it is degraded [63, 65]. We hypothesize *Ire1* upregulation in *Nr2e3* treated animals enhances UPR signaling activation, allowing the P23H rhodopsin protein to escape degradation in the ER and migrate to the outer segment of the retina as observed by IHC. However, whether or not the rescued rhodopsin in *Rho*^*P23H*^ mice upholds functional qualities has yet to be confirmed. Future studies include evaluating the role of *Ire1* in RP disease. Interestingly, when *Rho*^*P23H*^ mice were treated at P21, rhodopsin expression is not observed; however, blue and green opsin expression was rescued with AAV8-*Nr2e3* delivery at P0 or P21. Therefore, treating *Rho*^*P23H*^ with AAV8-*Nr2e3* at P21 may only rescue their cones. This underscores the potential of *Nr2e3* as a unique therapeutic agent to regulate complex genetic mechanisms in temporal and spatial manner.

Interestingly, our real time PCR results revealed that RP models showed very low to no expression of *Nr2e3* and other key transcription factors evaluated in this study (*Nrl, Crx, Trb, Rora*, and *Nr1d1*), which likely contributed to the progression of retinal degeneration in each model. Further, *Nr2e3* therapy increased the expression of these key transcription factors. This data suggest that *Nr2e3* action to restore photoreceptor development and improve retinal function occurs through the modulation of many gene networks and the recruitment of key photoreceptor transcription factors that work in concert to reset retinal homeostasis.

In summary, this study demonstrates the potency of a novel modifier gene therapy to elicit broad-spectrum therapeutic effects. Modifier genes, particularly NHRs that are capable of regulating numerous networks, can attenuate disease progression and strongly impact disease outcome. The results of this study illustrate that the attenuation of disease outcomes caused by mutations in different genes can be achieved with a single modifier gene. Future studies include in-depth analysis of photoreceptor function at the onset of disease for each model and at specific time points post treatment with *Nr2e3*. Future studies will also evaluate promoter specificity and potency, dosage studies, and combination therapies to determine optimal efficacy. Recent reports discuss the toxicity of using strong promoters such as CMV. However, this present study employs a CAG promoter and a dose fivefold less than the lowest dose evaluated recent studies [105, 106]. The results from this study can be translated to other human diseases and lay the foundation for the development of modifier gene therapy for other neurological diseases.

## Supplementary information

Supplemental Figure and Table Legends

Supplmental Figure 1

Supplmental Figure 2

Supplmental Table 1

Supplmental Table 2

Supplemental Table 3
