# Supplementary material for: Nr2e3 is a genetic modifier that rescues retinal degeneration and promotes homeostasis in multiple models of retinitis pigmentosa
Source: Gene Ther. 2020 Mar 2;28(5):223–41. doi: 10.1038/s41434-020-0134-z (PMC7483267; doi:10.1038/s41434-020-0134-z)
Supplement: Supplementary file 1 — Supplemental Figure and Table Legends [file 41434_2020_134_MOESM1_ESM.docx]

**Supplementary Figure 1. AAV8-*Nr2e3* vector map.**

A visual representation of the AAV2/8-*Nr2e3* vector, which consists of the *Nr2e3* gene under the control of CAG promoter and flanked by ITR.

**Supplementary Figure 2. AAV5-*Nr2e3*-GFP and AAV2.7m8-*Nr2e3* rescue of *rd7* clinical phenotype.**  **A.** Fundus and OCT of *rd7* animals before and 1 month after injected with AAV5-*Nr2e3*-GFP; injection at P90, evaluation at P120. GFP demonstrates localization of AAV5 to retina; OCT shows whorls in untreated and rescue in treated retinas **B.** Fundus and OCT of *rd7* animals before and 1 month after injected with AAV2.7m8-*Nr2e3*; injection at P30, evaluation at P60. OCT shows whorls in untreated and rescue in treated retinas. OCT whole retina images show frame location of each scan denoted by green line. Right panel of scans taken at the same frame before and one month after injection. Red arrows indicate whorls present in the scans before injection and resolved in scans one-month post injection (PI). N=5.

**Supplementary Table 1. Primer sequence of genes evaluated in treated and untreated retinas.** A total of 75 genes were evaluated in treated vs untreated RP retinas. The name and function of genes evaluated are shown in the table. Primers sequence for each gene is provided.

**Supplementary Table 2. Primer sequences for transcription factor genes**

**Supplementary Table 3. *Nr2e3* putative response elements (RE).**  Out of 75 genes tested, 19 genes were found to have one or more putative *Nr2e3* REs (three *Nr2e3* REs were found in *Erg* and two were found in *Ncor1,* other genes contain one *Nr2e3* RE). The name of genes containing putative *Nr2e3* REs; the sequence, length and location of putative *Nr2e3* REs found as well as the sequence of primers used in Chip-Real-time confirmation are listed in the table.
